# GenomeHubs: simple containerized setup of a custom Ensembl database and web server for any species

**DOI:** 10.1093/database/bax039

**Published:** 2017-05-15

**Authors:** Richard J. Challis, Sujai Kumar, Lewis Stevens, Mark Blaxter

**Affiliations:** Institute of Evolutionary Biology, The University of Edinburgh, Ashworth Laboratories, Charlotte Auerbach Road, Edinburgh, EH9 3FL, UK

## Abstract

As the generation and use of genomic datasets is becoming increasingly common in all areas of biology, the need for resources to collate, analyse and present data from one or more genome projects is becoming more pressing. The Ensembl platform is a powerful tool to make genome data and cross-species analyses easily accessible through a web interface and a comprehensive application programming interface. Here we introduce GenomeHubs, which provide a containerized environment to facilitate the setup and hosting of custom Ensembl genome browsers. This simplifies mirroring of existing content and import of new genomic data into the Ensembl database schema. GenomeHubs also provide a set of analysis containers to decorate imported genomes with results of standard analyses and functional annotations and support export to flat files, including EMBL format for submission of assemblies and annotations to International Nucleotide Sequence Database Collaboration.

**Database URL:** http://GenomeHubs.org

## Introduction

Access to genomic sequence data for target species now underpins a large component of research programmes across all areas of biology and ecology. This has fuelled rapid increases both in the number of genome-sequencing projects, and in the number of research groups generating, assembling and annotating draft genome sequences. In order to maximize the value of these data, it is important to ensure that they are made accessible to the widest possible community of users.

Academic publications require submission of draft genomes to the public databases (1) but often do not require submission of additional analyses such as gene prediction, functional annotation, variant-calling and orthology discovery. Although the results of these analyses are published, it can be hard to access the underlying data in consistent formats. Some researchers make these data available on their individual lab websites as downloadable files, but others make them more accessible by providing a genome browser, BLAST sequence search interface, and a way to find specific genes by name, orthology, function or protein domain annotation.

A number of tools have been developed to host and share genomic data, including GBrowse (2), JBrowse (3), UCSC Genome Browser (4), InterMine (5), PlantGenIE (6) and Ensembl (7). Ensembl is the most complete of these tools as it supports both single- and multi-species comparative views alongside variation and functional data. The term ‘Ensembl’ is colloquially used for the Ensembl websites hosted by the European Bioinformatics Institute (EBI). However, the Ensembl system that delivers these databases, developed and delivered by the EBI, includes a richly specified database schema as well as routines for data import and processing and a web codebase for interactive display of data. By using an Ensembl genome browser, projects can offer a familiar and standardized interface with access to a powerful application programming interface (API), and thus facilitate large-scale comparative analysis and data-mining. Genomic data also continue to be valuable well beyond the initial funding cycle. From an archival perspective, data imported to an Ensembl database are stored in a format that is likely to have long-term support with a mature database structure and codebase.

A small number of high-profile, taxonomically-focussed sites, such as VectorBase (8), WormBase ParaSite (9) AvianBase (10) and Gramene (11), have been set up using Ensembl. However despite the advantages of using the Ensembl web code, its adoption as a browser for more local genome project or lab-hosted databases has been limited. Ensembl has an extensive list of dependencies that must be installed before it can run and the complexity of the code and need to edit a number of interconnected configuration files can make it difficult to trace the cause of problems during installation.

Here we present GenomeHubs, a containerized approach to simplify setting up and importing data into custom Ensembl sites. GenomeHubs was developed to address some of the challenges of setting up the Lepidopteran genome database, Lepbase (12). GenomeHubs can be used to mirror any existing Ensembl species (i.e. a species already available at ensembl.org, including the Ensembl Genomes (13) sites), to import new sequence and gene model data into an Ensembl database from FASTA and General Feature Format (GFF) files, and to run and import additional analyses to provide functional annotations. Although initially developed for a taxon-oriented resource, we also present examples of project-based and lab-specific Ensembl instances that we have created using these pipelines and highlight the utility of GenomeHubs throughout the genome sequencing and assembly process.

## Materials and methods

### Containerization

GenomeHubs use containerization to facilitate the deployment and maintenance of a suite of tools and analysis software each with specific, occasionally incompatible, dependencies. Docker (www.docker.com) containers provide the flexibility to run tools in any environment that supports Docker Engine, from a personal computer through dedicated servers to deployment on a public or private cloud.

**Figure 1.**
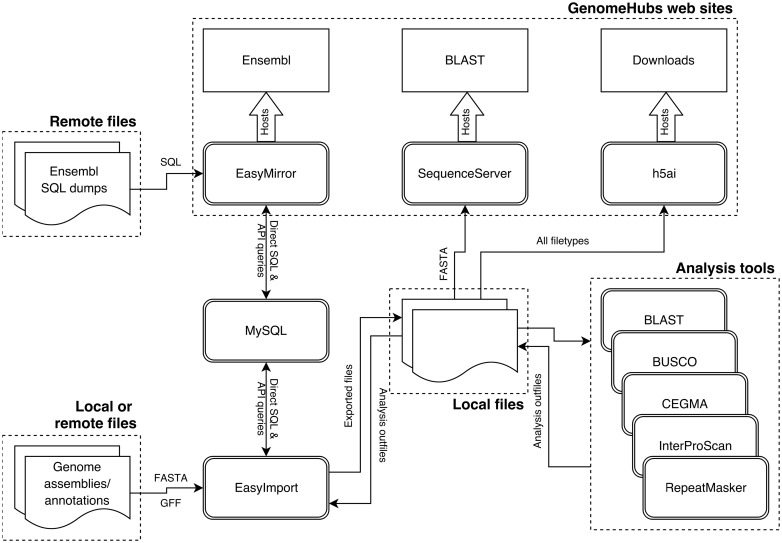
Schematic diagram of GenomeHubs containers and data flows.

Most of the containers used have been specifically created for this project (the exception being mysql-server) and can broadly be divided into three categories (Figure 1): (i) environments to run code developed as part of this project (EasyMirror and EasyImport); (ii) wrappers around existing web tools [h5ai file hosting server (https://larsjung.de/h5ai/), Sequenceserver BLAST server (14), and the Ensembl hosting component of EasyMirror]; and (iii) wrappers around existing analysis software used primarily for functional annotation of imported genome assemblies and gene models (CEGMA (15), BUSCO (16), RepeatMasker (17), InterProScan and BLAST against Swiss-Prot (18))

The EasyMirror and EasyImport containers provide a bridge between external data files and the Ensembl databases hosted in a MySQL container. The remaining containers act directly on files exported from Ensembl databases by EasyImport. GenomeHubs web services are hosted by the EasyMirror, SequenceServer and h5ai containers.

### Mirroring Ensembl

A basic GenomeHubs Ensembl mirror can be set up using only two containers: MySQL is required to host a session database along with any Ensembl data to be hosted locally, and EasyMirror is needed to set up local databases, generate the configuration files and run the Ensembl site. All the Ensembl sites hosted by the EBI provide MySQL database dumps that can be mirrored in GenomeHubs, but third-party database dumps can also be imported, provided they adhere to the Ensembl schema.

By default, EasyMirror runs three scripts: database.sh simplifies database and user setup, allowing data to be imported from remote database dumps and ensuring that database users are created with appropriate read/write or read-only permissions. The remaining scripts, update.sh and reload.sh are described below. A full GenomeHubs mirror also uses an h5ai container to host a downloads site and a Sequenceserver (14) container to host a BLAST server. The data for these additional tools are extracted from the locally hosted Ensembl databases by running export scripts in the EasyImport container.

Each of these containers can be customized by providing files in a configuration directory, which is mounted as a volume before the container’s startup script is run. Parameters for the EasyMirror and EasyImport containers are set using INI format configuration files, reflecting the use of INI files in Ensembl’s web code repository (ensembl-webcode). Our use of INI files enables users setting up GenomeHubs Ensembl instances to gain familiarity with the syntax conventions needed to further customize an instance. For h5ai and Sequenceserver, the configuration options include specifying an html file to be used as a masthead and custom css to provide site branding and links between the separate tools. Custom ruby scripts can be incorporated into Sequenceserver to control functions such as the generation of links from BLAST results to other resources (which is used in the GenomeHubs Sequenceserver container to provide links back to the GenomeHubs Ensembl browser), while h5ai and lighttpd configuration files can be replaced with custom versions in the h5ai container to control all settings offered by these programmes.

An example of a fully automated GenomeHubs mirroring script, with associated configuration files, is available at https://github.com/GenomeHubs/demo (also included in Supplementary Material S1). Depending on internet connection speed, this will set up a GenomeHubs Ensembl mirror of the Glanville fritillary (*Melitaea cinxia* (19)) genome assembly, and import a second assembly (of the winter moth, *Operophtera brumata* (20)) from FASTA and GFF, on an Ubuntu server running Docker in around one hour with no user intervention. This script (i) sets up a MySQL container; (ii) runs the EasyMirror database.sh script to create database users and creates a local copy of the melitaea_cinxia_core_32_85_1 database from the EnsemblGenomes ftp site; (iii) exports sequences and json files, and indexes the local database using an EasyImport container; (iv) starts up an h5ai container to host the exported files on a downloads site; (v) starts up a Sequenceserver container to provide a BLAST server; and (vi) starts up an EasyMirror container, running the update.sh script to set up Ensembl web code configuration files and the reload.sh script to start and host an Ensembl website. The second part of this script also automates importing a new genome assembly FASTA file and gene model GFF file into an Ensembl database as described in the next section.

Hosting an Ensembl site requires several Ensembl code repositories (hosted at https://github.com/ensembl and, optionally, at https://github.com/ensemblgenomes) and a large number of external dependencies, all of which we have preinstalled in the EasyMirror container. EasyMirror also uses the plugin architecture of ensembl-webcode to allow additional plugins to be cloned from alternate repositories. This allows custom plugins, including the GenomeHubs https://github.com/GenomeHubs/gh-ensembl-plugin, containing site-specific modifications to be loaded into an Ensembl site by the EasyMirror container. All git repositories are updated each time the update.sh script is run, pulling the latest changes from specified branches of each repository. Database connection parameters and basic species configuration files are written to files stored in the Ensembl https://github.com/ensembl/public-plugins/mirror repository. The reload.sh script is then run to start the web server and host the site at http://127.0.0.1:8081. This can be linked to a public domain name as required.

Files may be exported from the mirrored database using scripts within the EasyImport container to extract data via the Ensembl API. EasyImport scripts are called by passing flags to the container in the docker run command with a startup script to control details of filenames based on the supplied database parameter. Sequence files (scaffold, protein and coding sequences (CDS)) are exported to provide bulk data files for analysis, to provide file downloads (with the h5ai container) and to provide BLAST databases for sequence similarity searching (with the Sequenceserver container). Detailed summary statistics exported in JSON format are used in the assembly stats (21) and codon usage (22) visualizations.

Finally, the EasyImport script index_database.pl is used to index all sequence, feature and xref entries in a search/autocomplete database to replace the default direct MySQL search within ensembl-webcode.

### Importing to Ensembl

The EasyImport container also contains scripts to import new genome assemblies and their associated gene models from FASTA and GFF files. Files may be retrieved directly from local or remote directories by the summarize_files.pl script. The file locations are specified in an INI configuration file to ensure that the location of the source file used in the import is preserved to improve reproducibility. An example of an INI file and the commands used for importing a new genome assembly and annotation are given in Supplementary Material S1.

Files can be retrieved from any local directory or ftp/http server such as the European Nucleotide Archive (ENA) or NCBI ftp servers, individual lab web servers or sequencing provider servers. Subsequent steps will automatically retrieve required files so this step is optional. However it can be useful to fetch FASTA, GFF and additional annotation files for inspection prior to importing. If run, this step will report a summary of GFF attribute counts by feature type, which is particularly useful in determining which fields may contain IDs, descriptions, etc. and whether any discrepancies in the counts of each attribute suggest that the GFF needs to be repaired by the GFF parser.

### Database creation and sequence data import

The import_sequences.pl script includes wrappers around scripts already present in the Ensembl codebase: ensembl-production populate_production_db_tables.pl, and ensembl-pipeline load_seq_region.pl, load_agp.pl and set_top_level.pl scripts. Consistency with the Ensembl database schema is ensured by importing the database schema for each new core database from the Ensembl table.sql file and using an existing database with the same schema version as a template. Chromosome, scaffold and contig sequences are imported into the newly created database from the available FASTA and (optionally) AGP files.

### Parsing and repairing GFF

A significant challenge for a file-based approach to data import is dealing with the fragmentation of the GFF format and the diverse ways that names, IDs, descriptions and annotations can be captured both in GFF attributes and across a range of other file types. The EasyImport prepare_gff.pl script is centered around a flexible GFF parser (https://github.com/rjchallis/gff-parser) that is designed to embrace the diversity of real world GFF files by allowing full customization of expected relationships and properties with functions to repair, warn or ignore errors during validation.

This GFF parser is a perl module that provides a mechanism that assigns expectations and validation rules to specific GFF feature types while having very little hard-coded dependence on official gff specifications, giving flexibility to handle many more edge cases than parsers that have a strict requirement for valid input. This ensures that gene models and annotations can be extracted from diverse, often invalid GFF files. For EasyImport (in which the GFF parser is included as a git submodule) a subset of the full functionality can be controlled through a meta-syntax in the core import INI files. Although the GFF parser can accommodate departures from GFF specification, EasyImport requires a GFF3 compatible format in column nine (see Box 1). The flexibility of this approach to GFF parsing initially adds some complexity to parameter specification, but the benefit is that a record of all modifications to the original GFF3 file can be preserved in the INI file, maintaining a complete and unambiguous record of the import procedure to ensure full reproducibility. In order to reduce complexity, common patterns that can be reused across most GFF3 files are specified in a default.ini file that is automatically loaded in addition to the assembly specific INI file if present. 

### Gene model import

Although the GFF file format is repaired by the GFF parser, additional functionality in import_gene_models.pl allows for retrieval of gene IDs, synonyms and descriptions from GFF attributes as well as from FASTA headers and simple text files. EasyImport uses a simple match-and-replace syntax, specified in the INI file, to allow names to be matched across files where they may have different formats depending on whether they are gene, transcript or protein. For a community resource such as Lepbase, this provides the flexibility to incorporate information from diverse sources, as supplied by individual labs, rather than demanding a standardized format, which would act as a deterrent to full data sharing. For others implementing this pipeline, it offers the flexibility to integrate with existing protocols without the need to reformat data prior to import.

### Import verification

The most common problems with data import from GFF files can be detected through comparison of expected protein sequences with translations exported from an Ensembl database. verify_translations.pl checks that the same IDs are present in each of these sets, and that the sequences are identical. The most common causes of differences are alternate interpretations of phase and manual edits in the expected sequences file that terminate translations at the first stop codon. Typically, the import verification report is used to repair individual gene models or to edit the INI file to redefine the GFF feature expectations or naming rules before re-importing gene models.

### Functional annotation

Some xrefs can be imported via Dbxref attributes in a GFF file; however, we have deliberately limited the extent to which additional annotations can be imported from GFF due to the complexity of validating additional feature types and of mapping from potentially variable attribute names to specific fields in the Ensembl core database tables. Several xref types can be richly represented in the Ensembl database if all required attributes are provided and this is easiest to ensure by working directly with annotation software outputs.

EasyImport currently supports direct import of blastp (23), InterproScan (24), RepeatMasker (17), CEGMA (15) and BUSCO (16) output files and GenomeHubs containers and/or Dockerfiles are available to run each of these analyses with the appropriate parameters to generate the expected output filenames and formats. For Lepbase, we prefer performing these analyses independently using consistent software versions and parameter settings rather than using Dbxref attributes in the original GFF file as this helps to ensure consistency across genomes from diverse sources.

### Running Ensembl API scripts

The Ensembl website runs on top of the Ensembl API. As a result, the EasyMirror repository contains the perl repositories required to use the API, and any Ensembl API script may be run against locally hosted databases using the EasyMirror container with no further configuration. GenomeHubs thus makes it trivial to use the comprehensive range of scripts in the official Ensembl/EnsemblGenomes repositories. For example, Lepbase uses the Variant Effect Predictor (28) and import_vcf.pl scripts to set up and populate Ensembl Variation databases. 

## Results and Discussion

GenomeHubs facilitate the import of diverse data into Ensembl databases, making it relatively straightforward to set up and host Ensembl sites for any set of assemblies for which sequence data and gene models are available. Using the containerized approach described here, any bioinformatician comfortable with the command line will be able to set up a new GenomeHubs site within a few hours even if they have never encountered the Ensembl infrastructure before, and will then have access to the full suite of Ensembl web-based displays and comprehensive API for manipulating genomic data.

We currently use GenomeHubs to provide a taxon-oriented resource for published and pre-publication genomes at lepbase.org, and share draft genome assemblies and annotations at ensembl.caenorhabditis.org and ngenomes.org. Because it is now trivial to import even preliminary genomic resources into Ensembl, we have also begun to exploit the ease and power of the Ensembl API as part of the process of evaluation and improvement of draft genomes and annotations. 

### Taxon-oriented community resources

GenomeHubs were developed during the Lepbase (12) project to develop a taxon-oriented genomic resource for all lepidopteran (moths and butterflies) sequencing projects. Lepbase provides an Ensembl genome browser, an h5ai downloads server and a Sequenceserver BLAST server at lepbase.org.

As a taxon-oriented resource, Lepbase hosts genomes from a variety of sources with many different variations in file formats. Our initial species import scripts required modification to accommodate the idiosyncrasies of each new assembly dataset, which was an approach that scaled poorly as the number of available assemblies increased. For example, some genome providers supplied gene model information in Gene Transfer Format files that don’t model the parent–child relationships between CDS, mRNA and gene features. Other providers interpreted the ‘phase’ column (column 8) in GFF output in different ways, sometimes within the same file. Fortunately, EasyImport and the underlying gff-parser provide configuration options to deal with these and other variations, standardizing the import process, and speeding up the import of new genomes while allowing us to maintain a reproducible record of the process in the import INI file. Once imported, displaying new species data in our site only requires adding the new database name to the EasyMirror INI file.

Building and maintaining a community resource require orchestration of a diverse suite of tools and services. Moving each of the component tools, along with our analysis and import pipelines, into containers ensures that we are quickly able to replicate our setup while deploying new instances for development and testing and moving our site between servers.

From conversations with collaborators who generate genomic resources, we learned that a major obstacle to submitting DNA to the public databases (25) was the difficulty of formatting genome annotation files correctly for submission. GenomeHubs therefore supports export to an EMBL format file that can be deposited to the International Nucleotide Sequence Database Collaboration (INSDC), including the DNA Data Bank of Japan, ENA and GenBank databases.

### Sharing draft genome assemblies and annotations

The *Caenorhabditis* Genomes Project (CGP) aims to produce genome assemblies and annotations for all *Caenorhabditis* species (∼50) currently in culture. These data are shared between all project partners and made publicly available through the CGP Ensembl site at ensembl.caenorhabditis.org. This instance combines mirrored pre-existing Ensembl databases for several species (e.g. *C. elegans*) and custom databases based on imported data. This project-level genome browser is essential to the success of the CGP, allowing for the release of data which are often preliminary and hence not suitable for release at WormBase (26) and other public databases.

The Blaxter Lab is a genomics and bioinformatics group at the University of Edinburgh leading or collaborating on genomics projects across a large number of animals, plants, fungi and bacteria, which are hosted across a number of lab and project websites. The GenomeHubs pipelines provide an opportunity to rationalize many of these resources in a common interface. We have begun adding both published and unpublished projects to a lab GenomeHub setup at ensembl.ngenomes.org to accelerate the dissemination of our research and to enable collaborators to mine the data as they are released.

### Analysis of draft genomes

In addition to providing an environment for sharing draft genomes and their annotations, GenomeHubs can serve as a useful analysis tool both during and after the assembly process. A typical genome project will, over time, update and improve existing assemblies and add assemblies for other taxa. Analysing these data within GenomeHubs has several benefits. First, the import process acts to standardize the data, eliminating the issue of heterogenous file formats which complicate analysis pipelines. Secondly, the Ensembl Perl API supports a diverse range of functions, making the extraction of complex information from one or more genome assemblies trivial. Finally, scripts which extract information using the API can be reused on any other assembly or taxa, meaning that when additional genomes and/or new versions become available, the analysis can be easily repeated. An example script (Box 2) and its output (Table 1) are included, which were used to assess a predicted gene sets in versions of the *Ramazzottius varieornatus* genome (27) by summarizing the number of single exon genes with no InterPro domains as an indicator of prediction quality. The second version of the gene set showed a reduction of single exon genes from 15% to 5% of the total number of predicted genes, and increased content of protein domain annotations.

**Table 1.** Comparing the predicted gene sets of two versions of the *R. varieornatus* genome

	RVARI v1	RVARI v1.1
Total gene count	21 493	13 920
Single-exon genes	5340 (25%)	1711 (12%)
Single-exon genes with no structural domain hits	3251 (15%)	673 (5%)

An excess of single-exon genes, which are typically rare in eukaryotic genomes, can indicate poor quality gene predictions. RVARI v1 was found to have a high proportion of single-exon genes, the majority of which have no structural annotation, and are therefore likely erroneous. The gene set was re-predicted prior to analysis (27).

Box 1. Example syntax for GFF Parser configuration

GFF parser default patterns for creating valid exon and CDS features if missing

[GFF]

  CONDITION_1 = [MULTILINE CDS]

  CONDITION_2 = [LACKS_ID CDS make]

  CONDITION_3 = [EXPECTATION cds hasSister exon force]

  CONDITION_4 = [EXPECTATION cds hasParent mrna force]

   CONDITION_5 = [EXPECTATION exon hasParent transcript|mrna|mirna|trna|ncrna|rrna force]

   CONDITION_6 = [EXPECTATION mrna hasParent gene force]

Example GFF parser patterns for retrieving gene IDs, symbols, and descriptions from non-standard GFF attribute fields:

[GENE_STABLE_IDS]

 GFF = [gene->id/(.+)/]

[GENE_NAMES]

 GFF = [gene->symbol/(.+)/]

[GENE_DESCRIPTIONS]

 GFF = [1 DAUGHTER->product/(.+)/]

Example GFF parser pattern for obtaining a translation stable ID by replacing text in a transcript stable ID:

[TRANSLATION_STABLE_IDS]

 GFF = [SELF->ID/(.+)//-RA/-PA/]

Box 2. Example Ensembl Perl API script. This script retrieves all protein-coding genes from all scaffolds/contigs, finds the canonical transcript of each gene (which, for imported data, is the longest transcript) and counts the number of CDS elements. It then retrieves the amino acid translation of the transcript and counts the number of domains and features (which have been imported from an InterProScan annotation file). For each gene, it prints the transcript ID, the CDS count and the domain count. The full script is provided in Supplementary Material S2.

foreach my $slice (@{$supercontigs}) {

 my $genes = $slice->get_all_Genes;

 while (my $gene = shift @{$genes}) {

  next unless $gene->biotype() eq “protein_coding”;

  my $transcript = $gene->canonical_transcript();

  foreach my $CDS (@{$transcript->get_all_CDS()}) {$CDS_count ++;}

  my $protein = $transcript->translation();

  $domain_count = 0;

  foreach my $domains (@{$protein->get_all_ProteinFeatures()}) {

    $domain_count ++;

  }

  print $transcript->stable_id().“\t”.$CDS_count.“\t”.$domain_count.“\n”;

  $CDS_count = 0;

 }

}

## Future directions

### Networks of GenomeHubs

A key feature in the future development of GenomeHubs will be making full use of the Ensembl Compara database schema and API to provide a rich comparative resource for each group or research community that uses GenomeHubs for their taxa of interest. We have been actively working on data import to the Compara database schema, and have included over 12 000 gene trees in the latest (v4) release of Lepbase. This approach has allowed us the flexibility to implement alternative methods to the standard Ensembl Compara pipeline but is yet to be fully containerized. Unlike our approach to core species databases, the process of comparative analysis import is contingent on the analyses used and files generated so a full description of our comparative data import will be described separately alongside the analytical methods (Kumar *et al.*, in preparation).

We envisage that as more taxa are included in Ensembl genome browsers through GenomeHubs, individual hubs will increasingly be linked by shared taxa, whether included as focal species, reference assemblies or outgroups. Including these shared taxa in Ensembl Compara databases will create an opportunity to develop a network of GenomeHubs, providing a substantial resource for comparative analyses. Having the data in a standardized format and accessible through the Ensembl API will open up opportunities to discover phylogenetically independent contrasts and to test hypotheses developed in a single clade by running the same API scripts on additional clades for which suitable data are available.

### Tighter Ensembl integration

GenomeHubs have been developed independently of the EBI/Ensembl to address many of the challenges faced by the authors in setting up the Lepbase clade-level genomic resource. In developing GenomeHubs, we have reused Ensembl scripts and API functions but we have also frequently made the deliberate choice to write our own functions and to write scripts to operate directly on the MySQL databases, either to add functionality not available through the API or to implement functions in a way that was compatible with previous decisions to adopt alternate approaches. We are aware that this has led to some duplicated effort and future development will include a focus on reducing the redundancy between our code and the Ensembl codebase. GenomeHubs will need to continue to evolve to reflect changes made between Ensembl versions and this currency will be easier to maintain with more closely integrated code.

## Conclusions

Making genomic datasets available through a human- and machine-accessible online portal increases the value of those datasets to the wider community. GenomeHubs greatly simplify the process of setting up a custom Ensembl and adding new data, and are suitable for creating taxon-oriented, project-based and lab-specific Ensembl sites. This simplification makes it practical to use the Ensembl API during preliminary analyses of an assembly, allowing the same scripts to be run on subsequent assembly versions. Prior to publication, GenomeHubs provide export scripts to produce valid files for submission to INSDC. Post-publication, GenomeHubs maintain genome sequence, gene models and annotations in their correct contexts, supporting further analyses and data reuse. Containerization allows all stages of GenomeHubs setup, import and analysis to be run on any computer architecture and greatly reduces the time taken to set up the integrated suite of tools necessary for hosting and analysis. For users who do not wish to deploy all components and analyses using Docker, the availability of the Dockerfiles alongside all our source code at https://github.com/GenomeHubs/ensures that full details of our configuration are documented and can be replicated in an alternate system.

## Supplementary Material

Supplementary Data
