# Supplementary material for: GenomeHubs: simple containerized setup of a custom Ensembl database and web server for any species
Source: Database (Oxford). 2017 May 15;2017:bax039. doi: 10.1093/database/bax039 (PMC5467552; doi:10.1093/database/bax039)
Supplement: Supplementary Data [file bax039_Supp.zip › GenomeHubs-EnsemblResourcesSuppfile2 (1).pdf]

Supplementary File 2

The entire script to extract CDS and domain counts from the Ensembl Perl API (Box 2; Table 1).

```
#!/usr/bin/perl -w

use strict;
use Bio::EnsEMBL::DBSQL::DBAdaptor;

die "\nUsage: protein_domains.pl <database name>\n\n" unless $ARGV[0];

my $dbname = $ARGV[0];

my $dba = Bio::EnsEMBL::DBSQL::DBAdaptor->new(
    -dbname => $dbname,
    -host => 'localhost',
    -user => 'anonymous',
    -port   => '3306',
    -driver => 'mysql',
);

my $slice_adaptor    = $dba->get_adaptor("Slice");
my $supercontigs     = $slice_adaptor->fetch_all('toplevel');
my $domain_count     = 0;
my $CDS_count        = 0;

foreach my $slice (@{$supercontigs}) {
    my $genes = $slice->get_all_Genes;
    while ( my $gene = shift @{$genes} ) {
        next unless $gene->biotype() eq "protein_coding";
        my $transcript = $gene->canonical_transcript();
        foreach my $CDS (@{$transcript->get_all_CDS() } ) { $CDS_count++; }
        my $protein = $transcript->translation();
            $domain_count = 0;
             foreach my $domains (@{$protein->get_all_ProteinFeatures() } ) {
                   $domain_count++;
             }
        print $transcript->stable_id() . "\t" . $CDS_count . "\t" .
$domain_count . "\n";
        $CDS_count = 0;
    }
}
```
