# Supplementary material for: GenomeHubs: simple containerized setup of a custom Ensembl database and web server for any species
Source: Database (Oxford). 2017 May 15;2017:bax039. doi: 10.1093/database/bax039 (PMC5467552; doi:10.1093/database/bax039)
Supplement: Supplementary Data [file bax039_Supp.zip › GenomeHubs-EnsemblResourcesSuppfile1.pdf]

Supplementary File 1

Example GenomeHubs commands and configuration files to mirror and import into an Ensembl site. These files are included in the GenomeHubs demo repository (https://github.com/genomehubs/demo) and may be run to set up an example GenomeHubs site with the commands:

```
cd
git clone https://github.com/genomehubs/demo
demo/import.sh
```

Example script: import.sh

https://github.com/genomehubs/demo/blob/master/import.sh Sets up a GenomeHubs Ensembl/downloads/BLAST site with one species (the Glanville fritillary, *Melitaea cinxia*) mirrored from Ensembl Metazoa and a second species (the winter moth, *Opheroptera brumata*) imported directly from FASTA and GFF files.

```
#!/bin/bash

# ============================================================================
# import.sh - automated commands to import an assembly from FASTA and GFF
#
# Usage:
# cd
# git clone https://github.com/genomehubs/demo
# cd demo
# ./import.sh
#
# Prerequisites:
# Requires Docker and assumes UID 1000
# ============================================================================

echo Step 1. Set up mySQL container

docker run -d \
           --name genomehubs-mysql \
           -e MYSQL_ROOT_PASSWORD=rootuserpassword \
           -e MYSQL_ROOT_HOST='172.17.0.0/255.255.0.0' \
           mysql/mysql-server:5.5 &&

sleep 10 &&

echo Step 2. Set up template database using EasyMirror &&

docker run --rm \
           --name genomehubs-ensembl \
```

```
          -v ~/demo/genomehubs-import/ensembl/conf:/ensembl/conf \
          -v ~/demo/genomehubs-import/ensembl/logs:/ensembl/logs \
          --link genomehubs-mysql \
          -p 8081:8080 \
         genomehubs/easy-mirror:17.03 /ensembl/scripts/database.sh
/ensembl/conf/database.ini &&

echo Step 3. Import sequences, prepare gff and import gene models &&

docker run --rm \
          --name easy-import-operophtera_brumata_v1_core_32_85_1 \
          --link genomehubs-mysql \
          -v ~/demo/genomehubs-import/import/conf:/import/conf \
          -v ~/demo/genomehubs-import/import/data:/import/data \
          -v ~/demo/genomehubs-import/download/data:/import/download \
          -v ~/demo/genomehubs-import/blast/data:/import/blast \
          -e DATABASE=operophtera_brumata_v1_core_32_85_1 \
          -e FLAGS="-s -p -g" \
          genomehubs/easy-import:17.03 &&

echo Step 4. Export sequences, export json and index database for imported
Operophtera brumata &&

docker run --rm \
          --name easy-import-operophtera_brumata_v1_core_32_85_1 \
          --link genomehubs-mysql \
          -v ~/demo/genomehubs-import/import/conf:/import/conf \
          -v ~/demo/genomehubs-import/import/data:/import/data \
          -v ~/demo/genomehubs-import/download/data:/import/download \
          -v ~/demo/genomehubs-import/blast/data:/import/blast \
          -e DATABASE=operophtera_brumata_v1_core_32_85_1 \
          -e FLAGS="-e -j -i" \
          genomehubs/easy-import:17.03 &&

ls ~/demo/genomehubs-import/download/data/sequence/Operophtera* 2> /dev/null &&

echo Step 5. Export sequences, export json and index database for mirrored
Melitaea cinxia &&

docker run --rm \
          --name easy-import-melitaea_cinxia_core_32_85_1 \
          --link genomehubs-mysql \
          -v ~/demo/genomehubs-import/import/conf:/import/conf \
          -v ~/demo/genomehubs-import/import/data:/import/data \
          -v ~/demo/genomehubs-import/download/data:/import/download \
          -v ~/demo/genomehubs-import/blast/data:/import/blast \
          -e DATABASE=melitaea_cinxia_core_32_85_1 \
          -e FLAGS="-e -i -j" \
          genomehubs/easy-import:17.03 &&
```

```
ls ~/demo/genomehubs-import/download/data/sequence/Melitaea* 2> /dev/null &&

echo Step 6. Startup h5ai downloads server &&

docker run -d \
           --name genomehubs-h5ai \
           -v ~/demo/genomehubs-import/download/conf:/conf \
           -v ~/demo/genomehubs-import/download/data:/var/www/demo \
           -p 8082:8080 \
           genomehubs/h5ai:17.03 &&

echo Step 7. Startup SequenceServer BLAST server &&

docker run -d \
           --name genomehubs-sequenceserver \
           -v ~/demo/genomehubs-import/blast/conf:/conf \
           -v ~/demo/genomehubs-import/blast/data:/dbs \
           -p 8083:4567 \
           genomehubs/sequenceserver:17.03 &&

echo Step 8. Startup GenomeHubs Ensembl mirror &&

docker run -d \
           --name genomehubs-ensembl \
           -v ~/demo/genomehubs-import/ensembl/gh-conf:/ensembl/conf \
           -v ~/demo/genomehubs-import/ensembl/logs:/ensembl/logs \
           --link genomehubs-mysql \
           -p 8081:8080 \
           genomehubs/easy-mirror:17.03 &&

echo Step 9. Waiting for site to load &&

until $(curl --output /dev/null --silent --head --fail
http://127.0.0.1:8081//i/placeholder.png); do
    printf '.'
    sleep 5
done &&

echo done &&

echo Visit your mirror site at 127.0.0.1:8081 &&

exit

echo Unable to set up GenomeHubs site, removing containers

docker stop genomehubs-mysql && docker rm genomehubs-mysql
docker stop genomehubs-ensembl && docker rm genomehubs-ensembl
docker stop genomehubs-h5ai && docker rm genomehubs-h5ai
docker stop genomehubs-sequenceserver && docker rm genomehubs-sequenceserver
```

Database configuration file: database.ini

https://github.com/genomehubs/demo/blob/master/genomehubs-import/ensembl/conf/database.ini Specifies passwords for the database users required by Ensembl/genomehubs and contains instructions to download local copies of the ncbi_taxonomy and melitaea_cinxia_core_32_85_1 databases.

```
[DATABASE]
  DB_USER = anonymous
  DB_PASS =

  DB_SESSION_USER = sessionuser
  DB_SESSION_PASS = sessionuserpassword
  DB_SESSION_NAME = ensembl_accounts

  DB_IMPORT_USER = importuser
  DB_IMPORT_PASS = importuserpassword

  DB_ROOT_USER = root
  DB_ROOT_PASSWORD = rootuserpassword
  DB_PORT = 3306
  DB_HOST = genomehubs-mysql

[WEBSITE]
  ENSEMBL_WEBSITE_HOST = 172.17.0.0/255.255.0.0

[DATA_SOURCE]
  ENSEMBL_DB_URL = ftp://ftp.ensembl.org/pub/release-85/mysql/
  ENSEMBL_DB_REPLACE =
  ENSEMBL_DBS =

  EG_DB_URL = ftp://ftp.ensemblgenomes.org/pub/release-32/pan_ensembl/mysql/
  EG_DB_REPLACE =
  EG_DBS = [ ncbi_taxonomy ]

  SPECIES_DB_URL = ftp://ftp.ensemblgenomes.org/pub/release-32/metazoa/mysql/
  SPECIES_DB_REPLACE =
  SPECIES_DB_AUTO_EXPAND =
  SPECIES_DBS = [ melitaea_cinxia_core_32_85_1 ]

  MISC_DB_URL = ftp://ftp.ensembl.org/pub/release-79/mysql/
  MISC_DB_REPLACE =
  MISC_DBS = [ ensembl_accounts ]
```

EasyMirror configuration file: setup.ini

https://github.com/genomehubs/demo/blob/master/genomehubs-import/ensembl/conf/setup.ini Defines connection settings for the local database (inside a linked docker container) and remote databases used to retrieve data not hosted locally and lists databases and Ensembl plugins to include in the hosted site.

```
[DATABASE]
  DB_HOST = genomehubs-mysql
  DB_PORT = 3306
  DB_USER = anonymous
  DB_PASS =

  DB_SESSION_HOST = genomehubs-mysql
  DB_SESSION_PORT = 3306
  DB_SESSION_USER = sessionuser
  DB_SESSION_PASS = sessionuserpassword

  DB_FALLBACK_HOST = mysql-eg-publicsql.ebi.ac.uk
  DB_FALLBACK_PORT = 4157
  DB_FALLBACK_USER = anonymous
  DB_FALLBACK_PASS =

  DB_FALLBACK2_HOST = ensembldb.ensembl.org
  DB_FALLBACK2_PORT = 3306
  DB_FALLBACK2_USER = anonymous
  DB_FALLBACK2_PASS =

[REPOSITORIES]
  ENSEMBL_URL = https://github.com/Ensembl
  ENSEMBL_BRANCH = release/85

  BIOPERL_URL = https://github.com/bioperl
  BIOPERL_BRANCH = master

  GENOMEHUBS_PLUGIN_URL = https://github.com/genomehubs/gh-ensembl-plugin
  GENOMEHUBS_PLUGIN_BRANCH = 17.03
  GENOMEHUBS_PLUGIN_PACKAGE = EG::GenomeHubs

  EG_METAZOA_PLUGIN_URL = https://github.com/EnsemblGenomes/eg-web-metazoa
  EG_METAZOA_PLUGIN_BRANCH = release/eg/32
  EG_METAZOA_PLUGIN_PACKAGE = EG::Metazoa

  API_PLUGIN_URL = https://github.com/EnsemblGenomes/ensemblgenomes-api
  API_PLUGIN_BRANCH = release/eg/32
  API_PLUGIN_PACKAGE = EG::API

  EG_COMMON_PLUGIN_URL = https://github.com/EnsemblGenomes/eg-web-common
  EG_COMMON_PLUGIN_BRANCH = release/eg/32
```

```
  EG_COMMON_PLUGIN_PACKAGE = EG::Common

  PUBLIC_PLUGINS = [ ]

[WEBSITE]
  HTTP_PORT = 8080
  SERVER_ROOT = /ensembl

[DATA_SOURCE]
  SPECIES_DBS = [
  melitaea_cinxia_core_32_85_1
       operophtera_brumata_v1_core_32_85_1
       ]
  SPECIES_DB_AUTO_EXPAND [ ]
  MULTI_DBS = [ ensemblgenomes_ontology_32_85 ensemblgenomes_info_32
ensembl_archive_85 ensembl_website_85 ]
  COMPARA_DBS = [ ensembl_compara_metazoa_32_85
ensembl_compara_pan_homology_32_85 ]
```

EasyImport configuration file: opheroptera_brumata_core_32_85_1.ini

https://github.com/genomehubs/demo/blob/master/genomehubs-import/import/conf/operophtera_brumata_v1_core_32_85_1.ini Specifies assembly-specific settings: the database name to create and import into; metadata describing the species, assembly and genebuild; remote files to import; and patterns to obtain stable identifiers from the GFF file.

```
[DATABASE_CORE]
       NAME = operophtera_brumata_v1_core_32_85_1
[META]
       SPECIES.PRODUCTION_NAME = operophtera_brumata_v1
       SPECIES.SCIENTIFIC_NAME = Operophtera brumata
       SPECIES.COMMON_NAME = Winter moth
       SPECIES.DISPLAY_NAME = Operophtera brumata
       SPECIES.DIVISION = EnsemblMetazoa
       SPECIES.URL = Operophtera_brumata_v1
       SPECIES.TAXONOMY_ID = 104452
       SPECIES.ALIAS = [ operophtera_brumata operophtera_brumata_v1
operophtera%20brumata winter%20moth ]
       ASSEMBLY.NAME = v1
       ASSEMBLY.DATE = 2015-08-11
       ASSEMBLY.ACCESSION = GCA_001266575.1
       ASSEMBLY.DEFAULT = v1
       PROVIDER.NAME = Wageningen University
       PROVIDER.URL = http://www.bioinformatics.nl/wintermoth
       GENEBUILD.ID = 2
       GENEBUILD.START_DATE = 2016-08
```

```
	GENEBUILD.VERSION = 2
	GENEBUILD.METHOD = import
	SAMPLE.LOCATION_PARAM    = OBRU01_Sc00001:57580-69243
	SAMPLE.LOCATION_TEXT     = OBRU01_Sc00001:57580-69243
	SAMPLE.GENE_PARAM        = OBRU01_00004
	SAMPLE.GENE_TEXT         = OBRU01_00004
	SAMPLE.TRANSCRIPT_PARAM  = OBRU01_00004-RA
	SAMPLE.TRANSCRIPT_TEXT   = OBRU01_00004-RA
	SAMPLE.SEARCH_TEXT       = OBRU01_00015-RA
[FILES]
	SCAFFOLD = [ fa http://download.lepbase.org/v4/provider/Obru1.fsa.gz ]
	GFF = [ gff3 http://download.lepbase.org/v4/provider/Obru_genes.gff.gz ]
	PROTEIN = [ fa http://download.lepbase.org/v4/provider/ObruPep.fasta.gz ]
[GENE_STABLE_IDS]
	GFF = [ gene->Name /(.+)/ ]
[TRANSCRIPT_STABLE_IDS]
	GFF = [ SELF->Name /(.+)/ ]
[TRANSLATION_STABLE_IDS]
	GFF = [ SELF->Name /(.+)/ /-RA/-PA/ ]
[MODIFY]
	OVERWRITE_DB = 1
	TRUNCATE_SEQUENCE_TABLES = 1
	TRUNCATE_GENE_TABLES = 1
```

EasyImport configuration file: default.ini

https://github.com/genomehubs/demo/blob/master/genomehubs-import/import/conf/default.ini Sets common parameters as defaults to simplify the assembly-specific INI files. Any parameters in this file can be overwritten by entries in an assembly-specific INI file.

```
[DATABASE_CORE]
	HOST = genomehubs-mysql
	PORT = 3306
	RW_USER =
	RW_PASS =
	RO_USER = anonymous
	RO_PASS =
[DATABASE_SEARCH]
	NAME = genomehubs_search_32_85
	HOST = genomehubs-mysql
	PORT = 3306
	RW_USER = importuser
	RW_PASS = importuserpassword
	RO_USER = anonymous
	RO_PASS =
[DATABASE_TAXONOMY]
	NAME = ncbi_taxonomy
	HOST = genomehubs-mysql
	PORT = 3306
	RO_USER = anonymous
```

```
	RO_PASS =
[DATABASE_TEMPLATE]
	NAME = melitaea_cinxia_core_32_85_1
	HOST = genomehubs-mysql
	PORT = 3306
	RO_USER = anonymous
	RO_PASS =
[GFF]
	SORT = 1
	CHUNK = [ change region ]
	_CONDITION_1 = [ MULTILINE CDS ]
	_CONDITION_2 = [ MULTILINE cDNA_match ]
	_CONDITION_3 = [ MULTILINE match ]
	_CONDITION_4 = [ MULTILINE three_prime_utr ]
	_CONDITION_5 = [ MULTILINE five_prime_utr ]
	_CONDITION_6 = [ LACKS_ID  CDS make ]
	_CONDITION_7 = [ EXPECTATION cds  hasSister exon force ]
	_CONDITION_8 = [ EXPECTATION cds  hasParent mrna force ]
	_CONDITION_9 = [ EXPECTATION exon       hasParent
transcript|mrna|mirna|trna|ncrna|rrna force ]
	_CONDITION_10 = [ EXPECTATION mrna      hasParent gene force ]
	_CONDITION_11 = [ EXPECTATION ncrna hasParent gene force ]
	_CONDITION_12 = [ EXPECTATION trna      hasParent gene force ]
	_CONDITION_13 = [ EXPECTATION transcript        hasParent gene force ]
	_CONDITION_14 = [ EXPECTATION five_prime_utr hasParent mrna force ]
	_CONDITION_15 = [ EXPECTATION three_prime_utr hasParent mrna force ]
	_CONDITION_16 = [ EXPECTATION
cds|exon|mrna|five_prime_utr|three_prime_utr|transcript|trna|ncrna|gene
<=[_start,_end] SELF warn ]
```
